# Supplementary material for: A broadly neutralizing antibody protects Syrian hamsters against SARS-CoV-2 Omicron challenge
Source: Nat Commun. 2022 Jun 23;13:3589. doi: 10.1038/s41467-022-31259-7 (PMC9223271; doi:10.1038/s41467-022-31259-7)

Supplementary Information

A broadly neutralizing antibody protects Syrian hamsters against SARS-CoV-2 Omicron challenge

Authors:

Biao Zhou[1,2,12], Runhong Zhou[1,2,12], Bingjie Tang[3,12], Jasper Fuk-Woo Chan[2,4,5,6,7,12], Mengxiao Luo[1,2,12], Qiaoli Peng[1,2,8,12], Shuofeng Yuan[2,4,5,6,7,12], Hang Liu[3,12], Bobo Wing-Yee Mok[2,4,5], Bohao Chen[1,2], Pui Wang[2,4,5], Vincent Kwok-Man Poon[2], Hin Chu[2,4,5], Chris Chung-Sing Chan[2], Jessica Oi-Ling Tsang[2], Chris Chun-Yiu Chan[2], Ka-Kit Au[1,2], Hiu-On Man[1,2], Lu Lu[2], Kelvin Kai-Wang To[2,4,5,6,7], Honglin Chen[2,4,5,6], Kwok-Yung Yuen[2,4,5,6,7,9], Shangyu Dang[3,10,11*] and Zhiwei Chen[1,2,4,5,6*]

Affiliations:

[1]AIDS Institute, Li Ka Shing Faculty of Medicine, The University of Hong Kong, Pokfulam, Hong Kong Special Administrative Region, People's Republic of China.

[2]Department of Microbiology, Li Ka Shing Faculty of Medicine, The University of Hong Kong, Pokfulam, Hong Kong Special Administrative Region, People's Republic of China.

[3]Division of Life Science, The Hong Kong University of Science and Technology, Clear Water Bay, Kowloon, Hong Kong Special Administrative Region, People's Republic of China.

[4]State Key Laboratory of Emerging Infectious Diseases, The University of Hong Kong, Pokfulam, Hong Kong Special Administrative Region, People's Republic of China.

[5]Centre for Virology, Vaccinology and Therapeutics, Health@InnoHK, The University of Hong Kong, Hong Kong Special Administrative Region, Hong Kong, People's Republic of China.

[6]Department of Clinical Microbiology and Infection Control, The University of Hong Kong-Shenzhen Hospital, Shenzhen, Guangdong, People's Republic of China.

[7]Department of Microbiology, Queen Mary Hospital, Pokfulam, Hong Kong Special Administrative Region, People's Republic of China.

[8]National Clinical Research Center for Infectious Diseases, HKU-AIDS Institute Shenzhen Research laboratory, The Third People's Hospital of Shenzhen, The Second Affiliated Hospital of Southern University of Science and Technology, Shenzhen, Guangdong, People's Republic of China.

[9]Academician Workstation of Hainan Province and Hainan Medical University-The University of Hong Kong Joint Laboratory of Tropical Infectious Diseases, The University of Hong Kong, Pokfulam, Hong Kong Special Administrative Region, People's Republic of China.

[10]Southern Marine Science and Engineering Guangdong Laboratory, Guangzhou, People's Republic of China.

[11]Center of Systems Biology and Human Health, Hong Kong University of Science and Technology, Clear Water Bay, Kowloon, Hong Kong Special Administrative Region, People's Republic of China.

[12]These authors contributed equally.

*Correspondence to: zchenai@hku.hk (lead contact) and sdang@ust.hk

Supplementary information

Supplementary Figures 1-7

Supplementary Tables 1-8

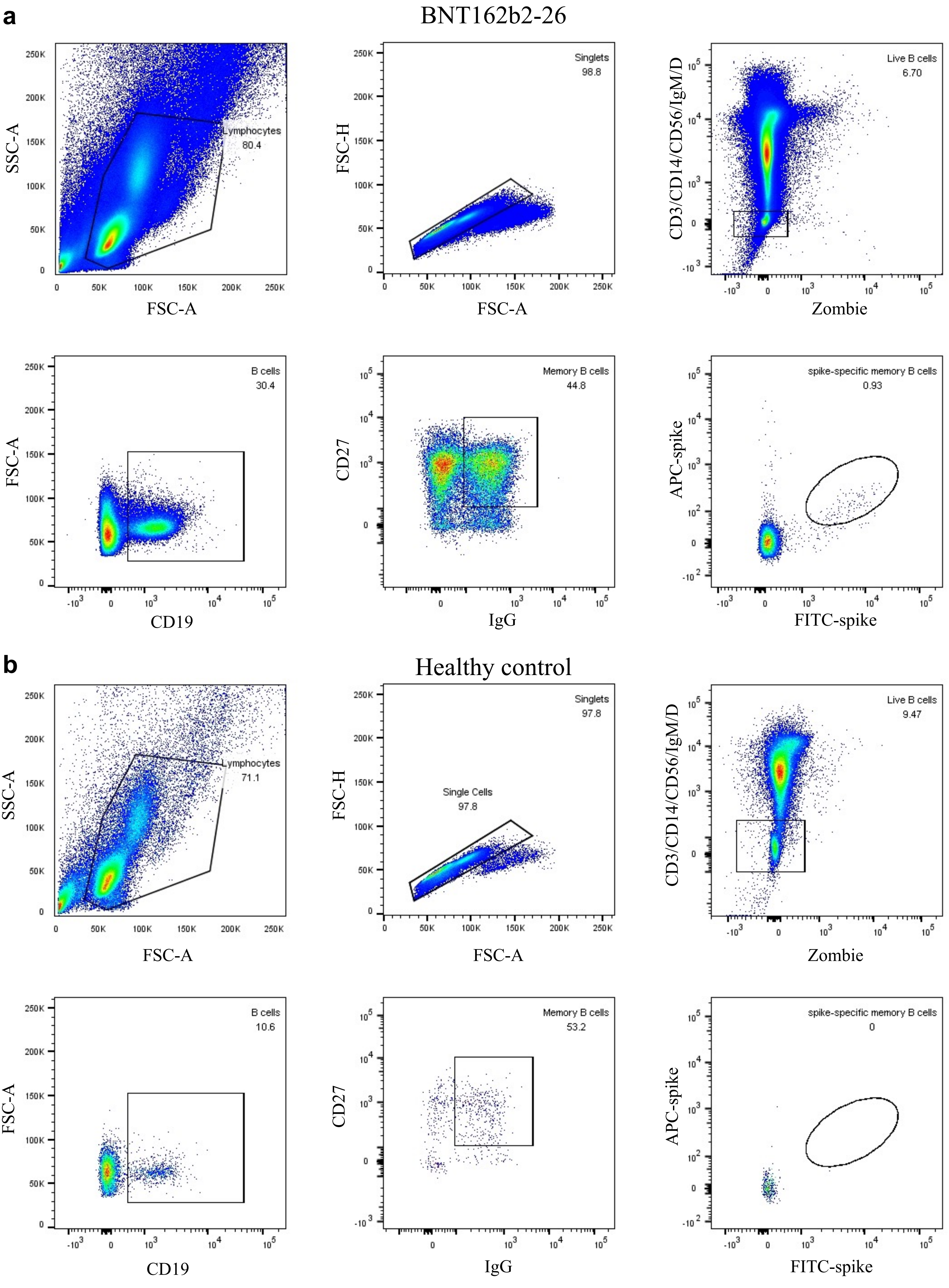

Supplementary Fig. 1 Gating strategy for sorting antigen specific memory B cells from the BNT162b2-26 vaccinee (a) as compared with a healthy control (b).

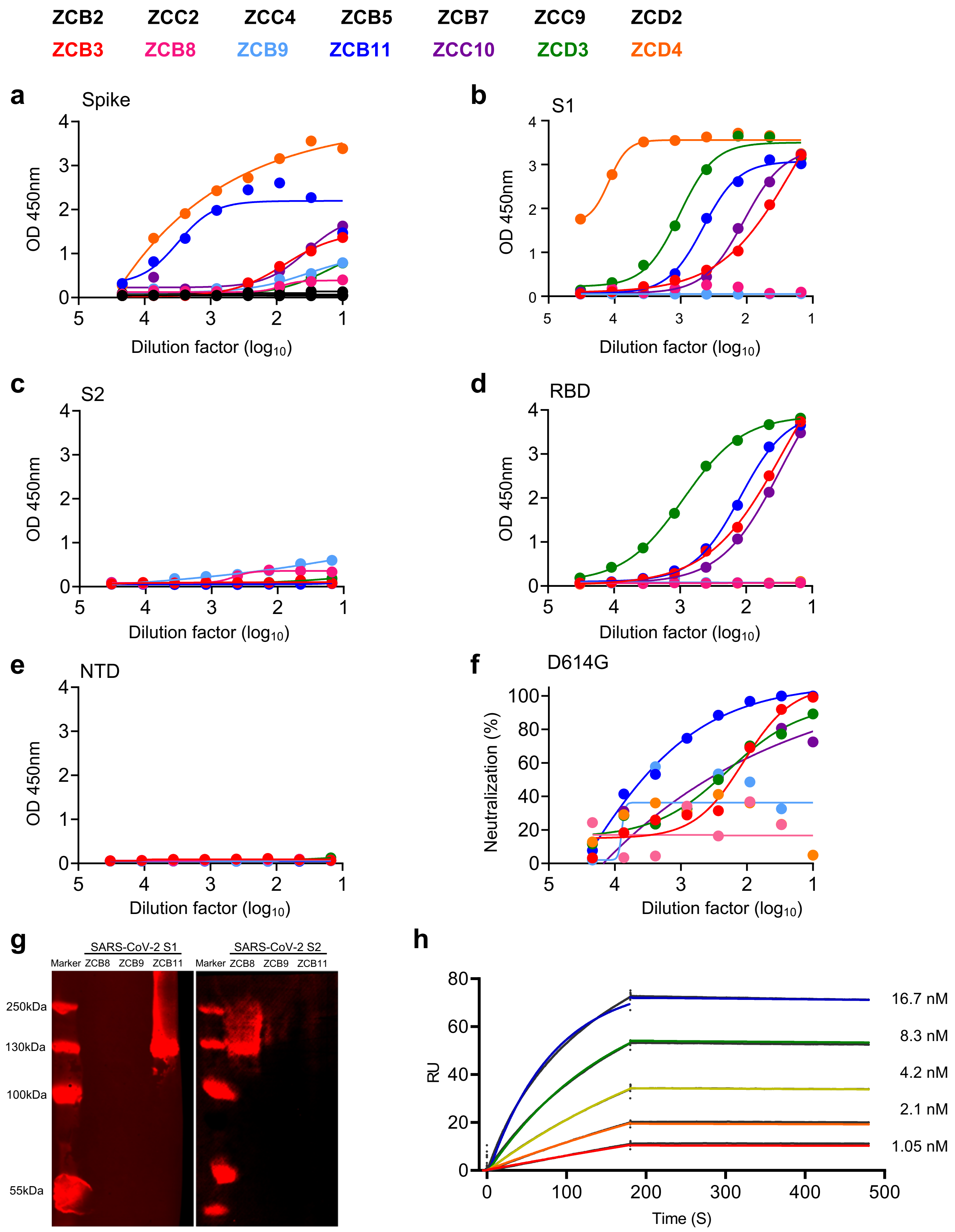

Supplementary Fig. 2 Binding and neutralizing activities of 14 newly cloned human monoclonal antibodies. (a-e) Culture supernatants containing expressed monoclonal antibodies were subjected to binding test to SARS-CoV-2 Spike (**a**), S1 (**b**), S2 (**c**), RBD (**d**) and NTD (**e**) by ELISA, respectively. (**f**) Neutralization activities of culture supernatants containing expressed antibodies were determined by the pseudotyped SARS-CoV-2 WT in 293T-ACE2 cells. (**g**) Western blotting to test the epitopes of ZCB8 and ZCB9. The antibodies were blotted with SARS-CoV-2 S1 (left) or S2 (right) after gel running and transferring. (**h**) Multiple-cycle binding kinetics of ZCB11 by SPR. SARS-CoV-2 RBD was immobilized onto a sensor chip followed by injection of purified ZCB11 at five different concentrations. The black lines indicated the experimentally derived curves while the color lines represented fitted curves based on the experimental data. Source data are provided as a Source Data file.

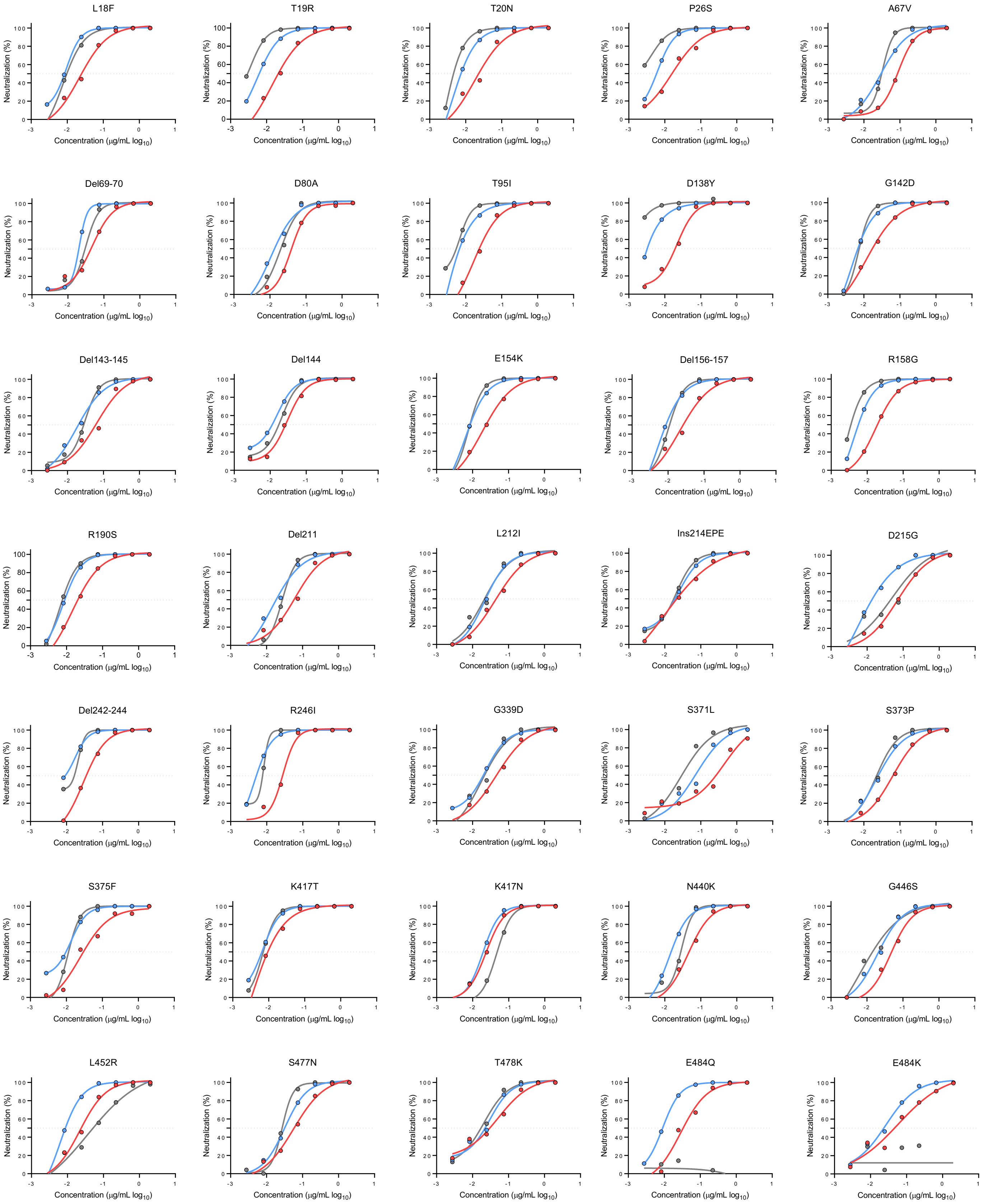
L18F
T19R
T20N
P26S
A67V
Del69-70
D80A
T95I
D138Y
G142D
Del143-145
Del144
E154K
Del156-157
R158G
R190S
Del211
L212I
Ins214EPE
D215G
Del242-244
R246I
G339D
S371L
S373P
S375F
K417T
K417N
N440K
G446S
L452R
S477N
T478K
E484Q
E484K
Neutralization (%)
Concentration (μg/mL log10)

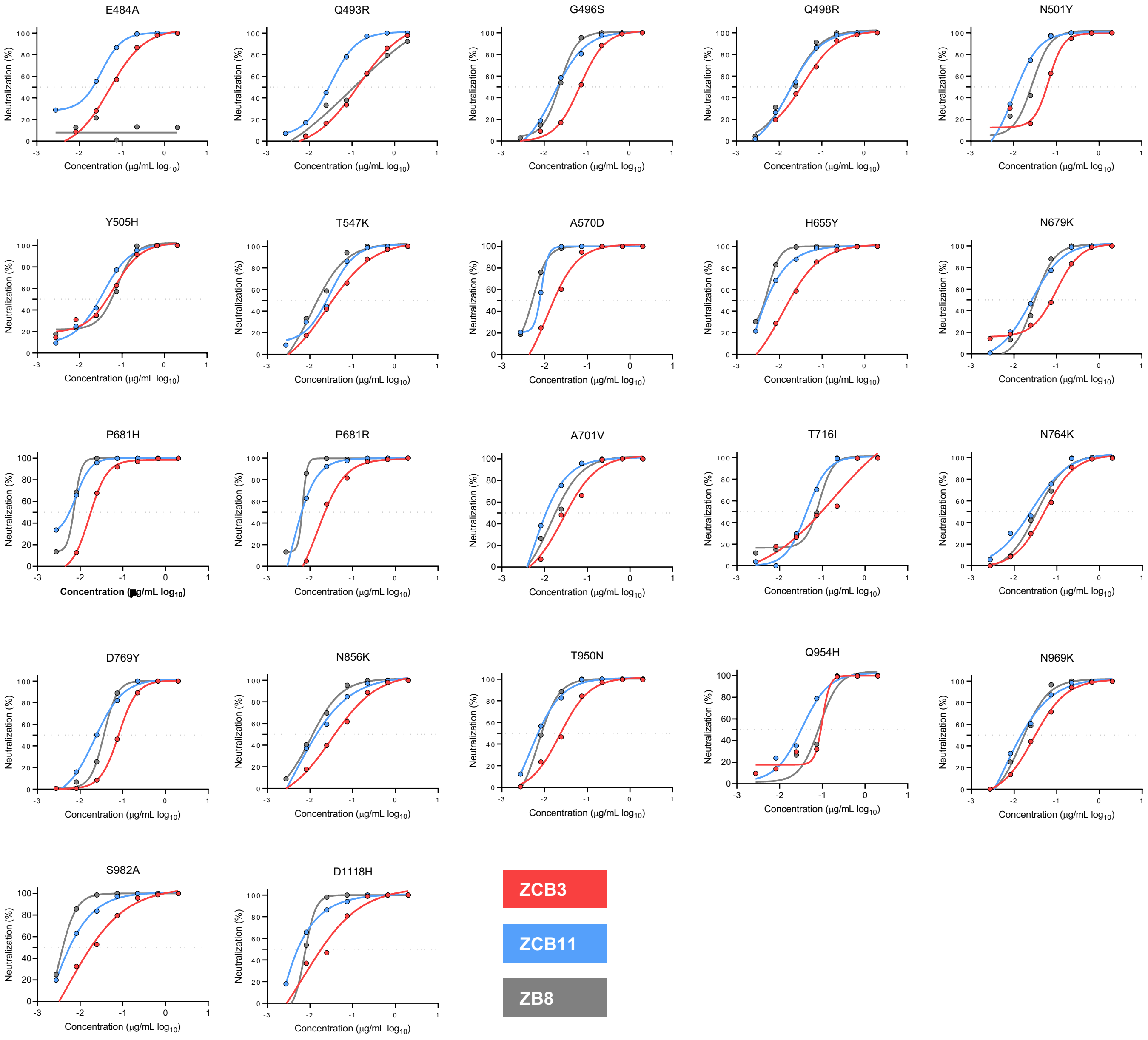

Supplementary Fig. 3 Neutralization against SARS-CoV-2 pseudoviruses carrying single mutation naturally occurring in SARS-CoV-2 VOCs. The neutralization assays against pseudoviruses carrying single mutation were performed to test the potency change of ZCB3 (red), ZCB11(blue) and ZB8 (grey). The color coding was consistently used in each graph. Source data are provided as a Source Data file.

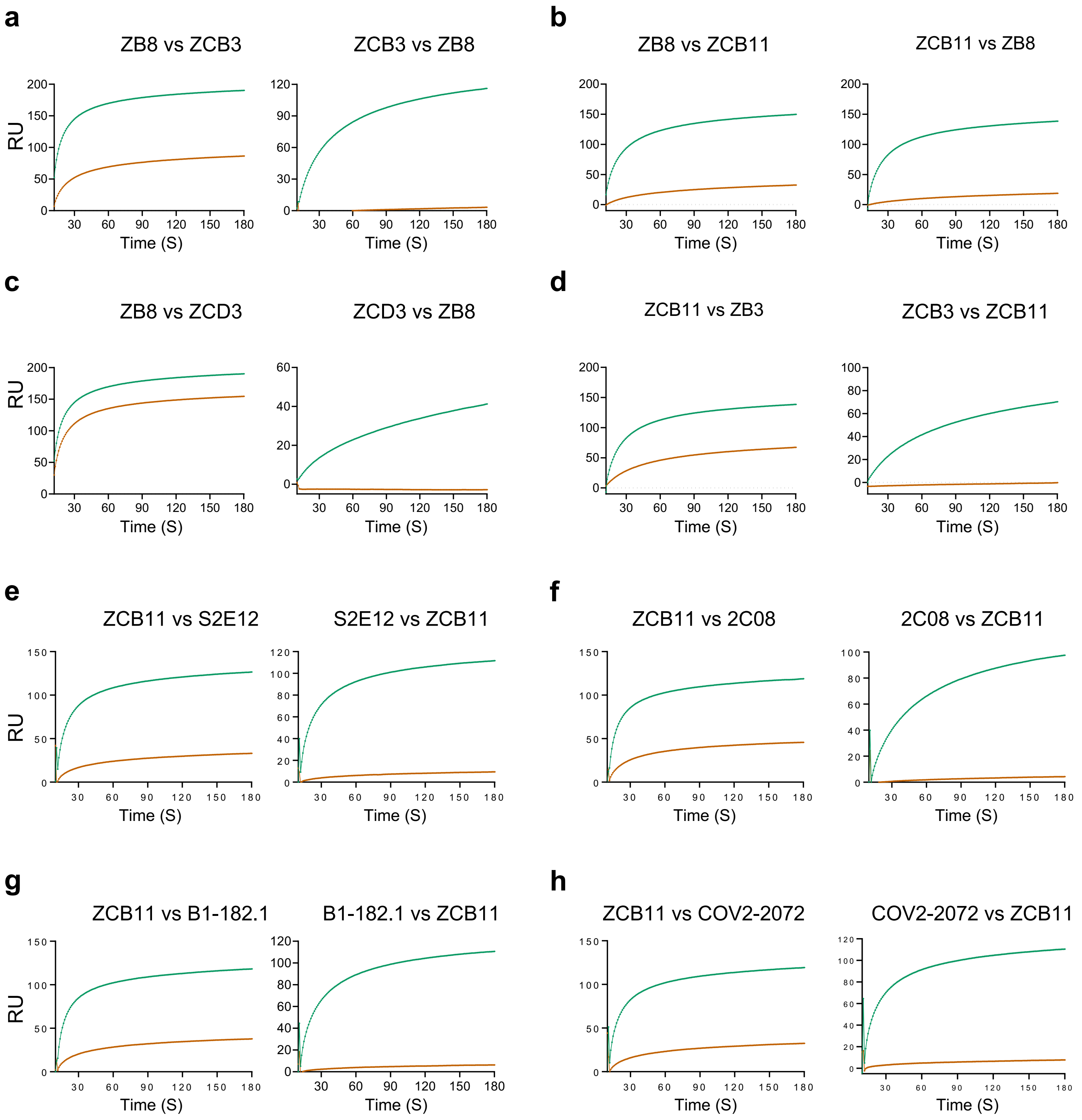

Supplementary Fig. 4 Competitions between ZCB11 and known antibodies for binding to SARS-CoV-2 RBD. (**a-d**) The competitions between ZB8 and newly isolated antibodies in this study. (**e-h**) The competitions between ZCB11 and public antibodies with VH1-58 clonotypes isolated by other groups. The sensorgrams show distinct binding patterns when pairs of testing antibodies were sequentially applied to the immobilized SARS-CoV-2 RBD. Color coding curves indicate distinct binding patterns of representative NAbs to RBD with (orange) or without (green) prior incubation with each testing antibody. Source data are provided as a Source Data file.

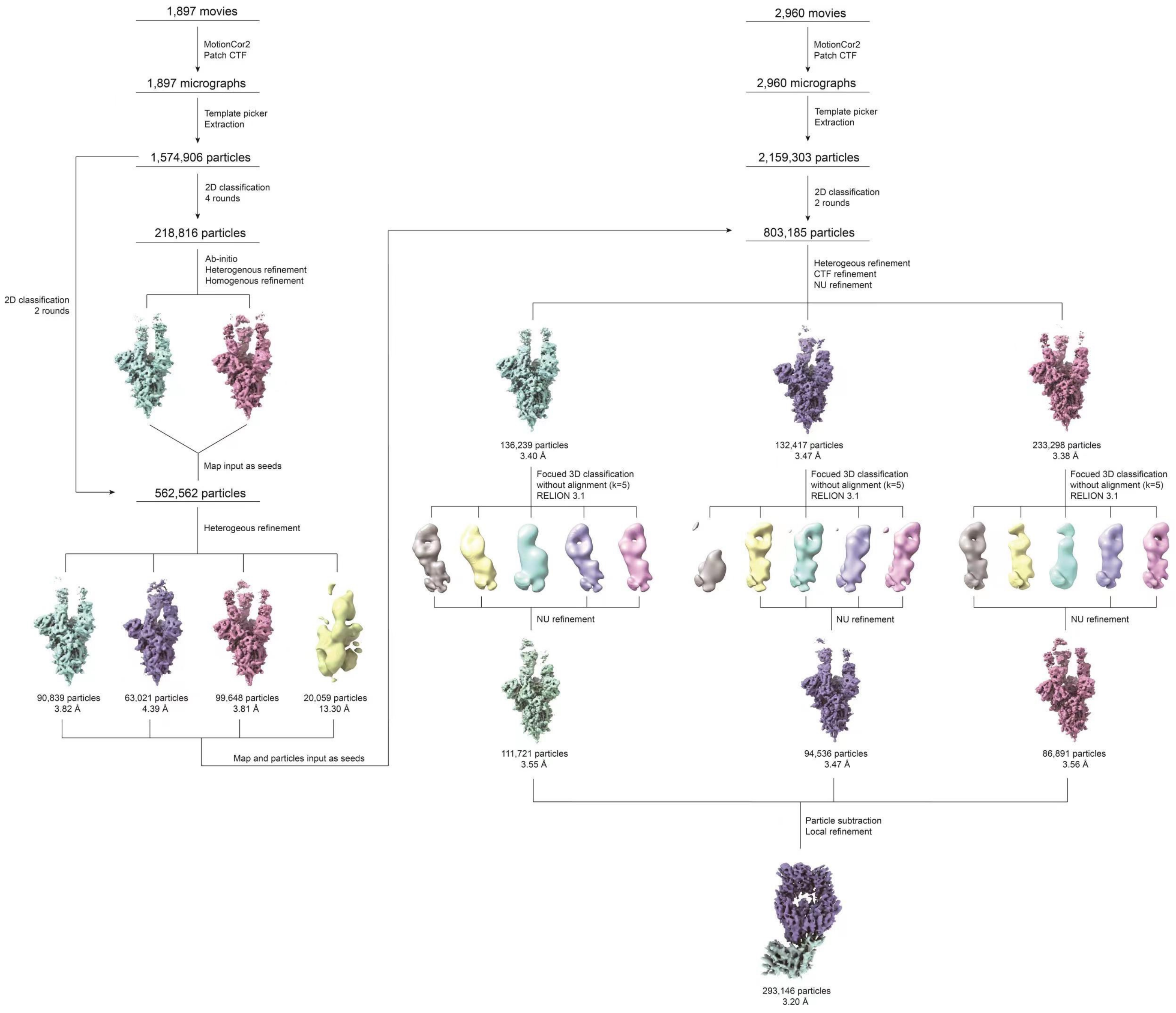

Supplementary Fig. 5. Cryo-EM Data processing of spike-Fab complex. Number of particles and the reconstruction resolution is indicated at every step.

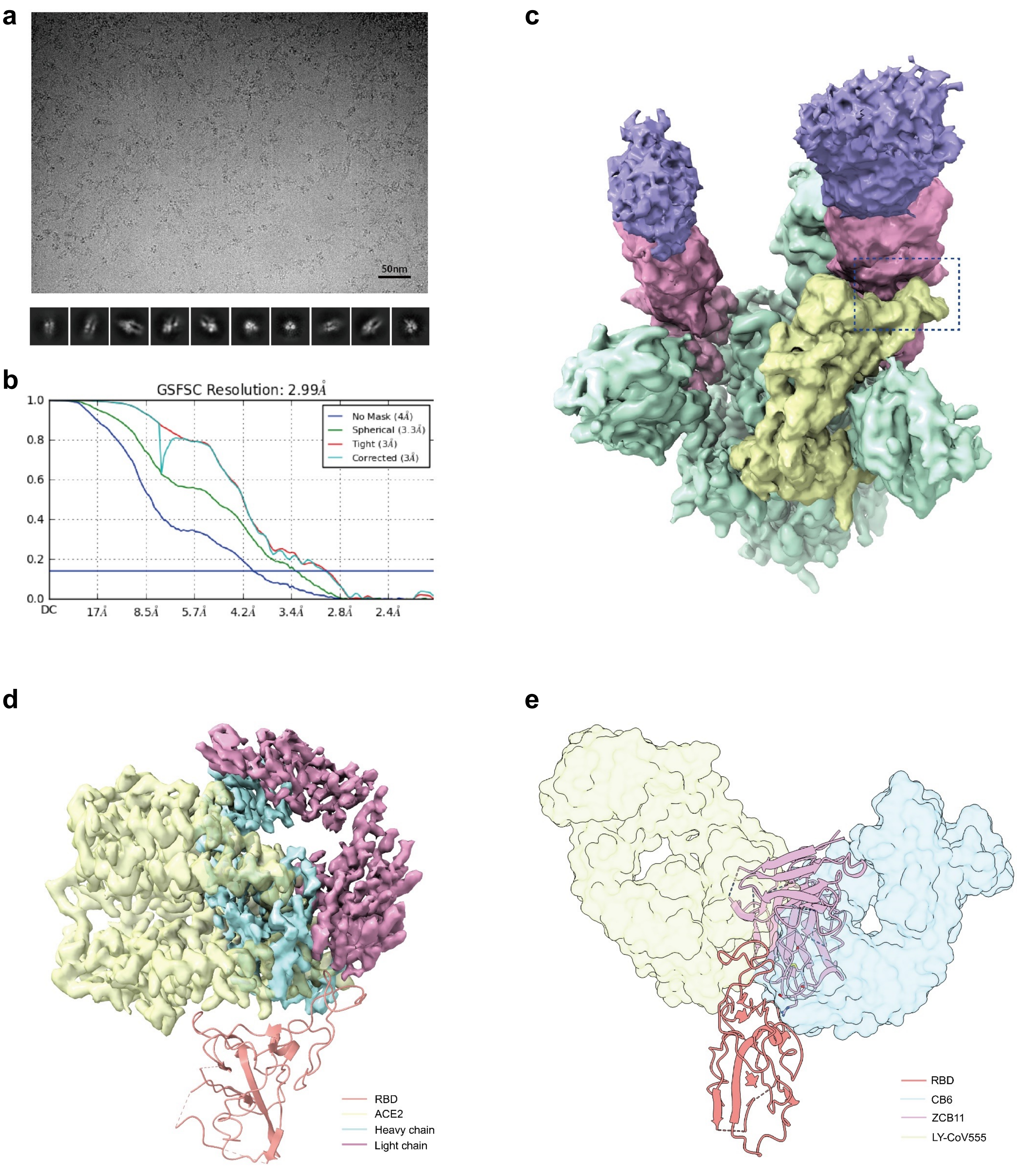

Supplementary Fig. 6. Cryo-EM analysis of spike-Fab complex. (**a**) A representative cryo-EM micrograph (n=4857) of spike-Fab complex. Featured 2D-class averages are shown. (**b**) Gold-standard Fourier shell correlation (FSC) curves of the final local refinement density map of RBD-ZCB11 interface. (**c**) Cryo-EM density map of 2u1d spike-Fab complex shows no Fab bound on the RBD in down conformation from the top view. Region of RBM interacting with ZCB11 is indicated. (**d**) Comparison between the binding patterns of ZCB11 (heavy chain density map: blue; light chain density map: pink) and ACE2 density map (yellow) with RBD (PDB backbone: orange). (**e**) Comparison between the binding patterns of ZCB11 (violet), CB6 (blue) (PDB: 7C01[https://www.rcsb.org/structure/7C01]) and LY-CoV555 (yellow) (PDB: 7L3N[https://www.rcsb.org/structure/7L3N]). CB6 and LY-CoV555 are shown as density maps.

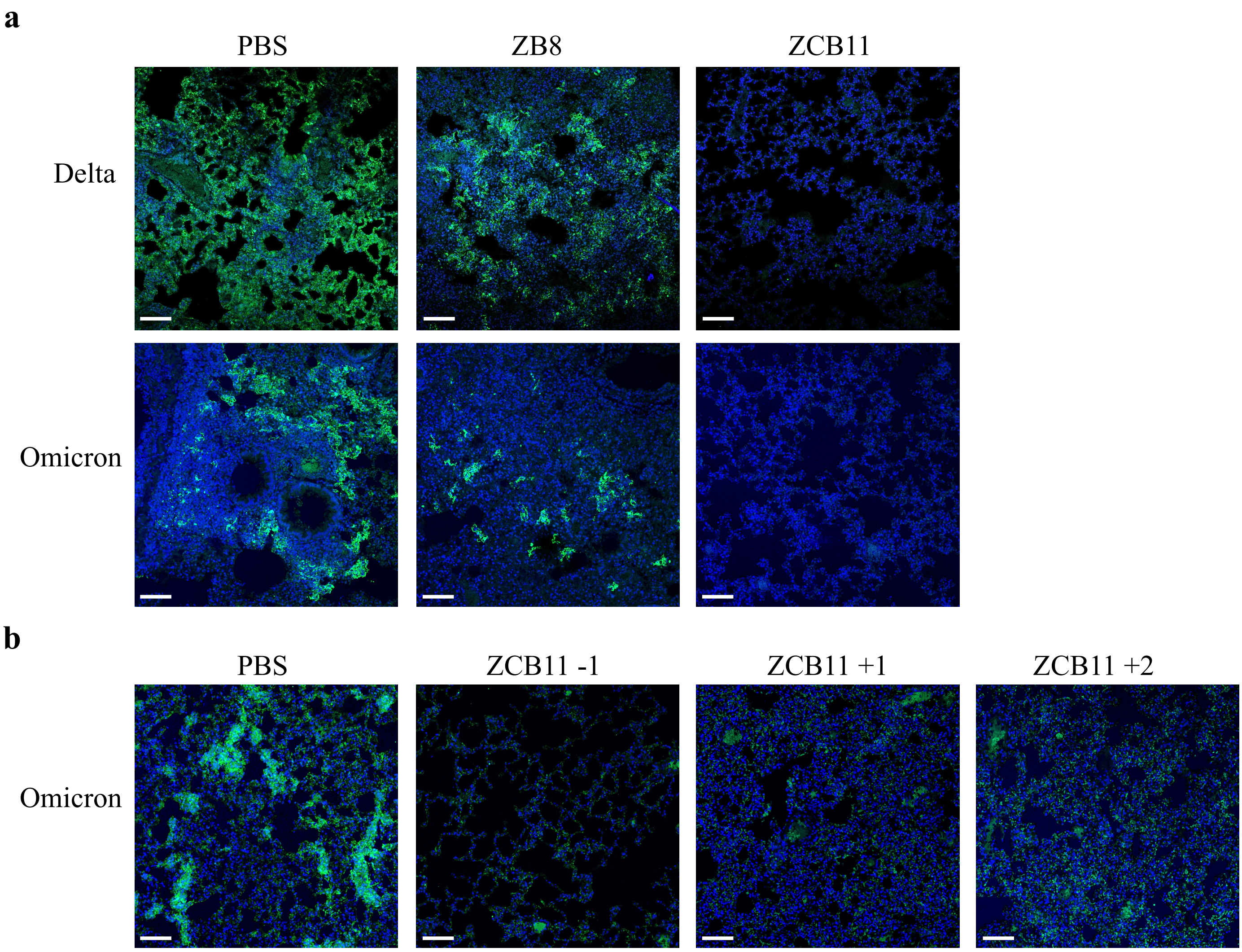

Supplementary Fig. 7 SARS-CoV-2 infection at 4 dpi in lungs of infected hamsters pre-treated or post-treated with neutralizing antibodies by confocal microscope. (**a-b**) Representative images (100×) of infected SARS-CoV-2 in lungs from each group of hamsters (n=4) as indicated. The SARS-CoV-2 strain used to infect hamsters was listed on the left and the treatment was shown on the top. Viral nucleocapsid protein (NP) was stained in green by immunofluorescence staining and cell nuclei were stained in blue with DAPI. The scale bar represents 100μm.

Supplementary Table 1. Characteristics of BNT162b2 vaccinees.

Vaccinee ID	Age	Gender	Vaccination doses	Sample collection (days post 2nd vaccination)
BNT162b2-1	28	F	2	30
BNT162b2-2	29	M	2	31
BNT162b2-3	31	M	2	31
BNT162b2-4	22	M	2	30
BNT162b2-5	36	M	2	31
BNT162b2-6	32	M	2	31
BNT162b2-11	26	M	2	24
BNT162b2-12	27	F	2	31
BNT162b2-13	35	F	2	24
BNT162b2-16	25	F	2	31
BNT162b2-18	29	F	2	31
BNT162b2-19	30	M	2	30
BNT162b2-20	27	F	2	30
BNT162b2-21	52	F	2	31
BNT162b2-24	35	F	2	22
BNT162b2-25	35	M	2	22
BNT162b2-26	58	M	2	7
BNT162b2-27	29	F	2	26
BNT162b2-28	25	M	2	26
BNT162b2-35	25	F	2	14
BNT162b2-37	33	F	2	47
BNT162b2-38	20	M	2	47
BNT162b2-39	32	F	2	25
BNT162b2-42	32	M	2	47
BNT162b2-46	25	M	2	42
BNT162b2-55	25	M	2	12
BNT162b2-57	52	M	2	18
BNT162b2-58	47	F	2	11
BNT162b2-59	44	F	2	17
BNT162b2-62	30	F	2	37
BNT162b2-63	29	F	2	47
BNT162b2-64	66	F	2	36
BNT162b2-67	50	F	2	12
BNT162b2-68	33	M	2	43

Supplementary Table 2. Neutralization titers of BNT162b2-26 plasma.

Sample	Neutralization titers (IC_{50})					
	WT	Alpha	Beta	Gamma	Delta	Omicron
BNT162b2-26	899	982	5085	466	2229	115
Average	731	474	95	532	164	35

Supplementary Table 3. Epitopes of isolated SARS-CoV-2 specific antibodies.

Antibody	Epitope
ZCB3	RBD
ZCB8	S2
ZCB9	S
ZCB11	RBD
ZCC10	RBD
ZCD3	RBD
ZCD4	S1

Supplementary Table 4. Binding ability of public NAbs to SARS-CoV-2 RBD and spike.

NAbs	SARS-CoV-2 RBD	SARS-CoV-2 spike
	EC_{50} (μg/mL)	EC_{50} (μg/mL)
ZCB3	0.027	0.092
ZCB11	0.020	0.020
ZCC10	0.041	0.245
ZCD3	0.156	1.582

Supplementary Table 5. Surface plasmon resonance analysis of ZCB11.

Curve	Conc (M)	ka (1/Ms)	kd (1/s)	KD (M)	Rmax (RU)	tc
0.15625 μg/mL	1.04E-09					
0.3125 μg/mL	2.08E-09					
0.625 μg/mL	4.17E-09					
1.25 μg/mL	8.33E-09					
2.5 μg/mL	1.67E-08					
		7.35E+05	4.22E-05	5.75E-11	81.21	9.11E+14

Supplementary Table 6. Neutralization IC_{50} values of public NAbs.

NAbs	Pseudovirus IC_{50} (ng/mL)						Live virus IC_{50} (ng/mL)					
	WT	α	β	γ	Δ	o	WT	α	β	γ	Δ	o
ZCB3	40.7	16.1	57.7	37.8	77.9	531.6	176.3	312.5	1383	540.6	41.3	6450
ZCB11	5.2	8.9	6.1	34.5	31.5	6	51	85.1	39.9	56.9	11.2	36.8
ZCC10	316.2	60.5	*N.A.*	1353	369.7	*N.A.*	/	/	/	/	/	/
ZCD3	355.8	210.1	*N.A.*	344.7	141.2	*N.A.*	2358	*N.A.*	*N.A.*	*N.A.*	392.6	*N.A.*

N.A.: Not applicable

Supplementary Table 7. Gene family analysis of four neutralizing antibodies.

NAbs	Heavy chain				Light chain			
	IGHV	IGHJ	CDR3 length	SHM (%)	IGKV	IGKJ	CDR3 length	SHM (%)
ZCB3	IGHV3-53*04	IGHJ6*02	12	5.1	IGKV1-9*01	IGKJ2*01	9	3.2
ZCB11	IGHV1-58*02	IGHJ3*02	16	5.5	IGKV3-20*01	IGKJ1*01	9	2.8
ZCC10	IGHV3-53*04	IGHJ6*02	12	5.1	IGKV3-20*01	IGKJ4*01	8	1.7
ZCD3	IGHV3-66*01	IGHJ4*02	16	3.8	IGKV1-27*01	IGKJ1*01	10	1.4

Supplementary Table 8. Statistics of cryo-EM data collection, processing and model refinement.

**Data collection**	
EM equipment	Titan Krios
Voltage(kV)	300
Detector	Gatan K3 summit
Magnification	81,000x
Electron dose (e/Å²)	50
Defocus range (μm)	-1.0~-2.5
Pixel size (Å)	1.06
Collected movies	4,857
**Reconstruction**	
Software	cryoSPARC v 2.15.0, RELION 3.1
Final particles	293,146
B-factors (Å^2)	-71.4
Map resolution (Å)	2.99(0.143)
**Atomic modelling**	
Software	UCSF Chimera 1.15, ChimeraX 1.3, Coot 0.9.5, Phenix 1.20
Chain	3
Residues	404
Water	0
Atoms	3164 (Hydrogens:0)
RMSD Length (Å)	0.014
RMSD Angles (Å)	1.894
**Ramachandran plot (%)**	
Favoured	95.48
Allowed	3.52
Outliers	1.01
Rotamer outliers	0.88
C-beta outliers	0

Supplementary Fig. 2g uncropped scans of western blots

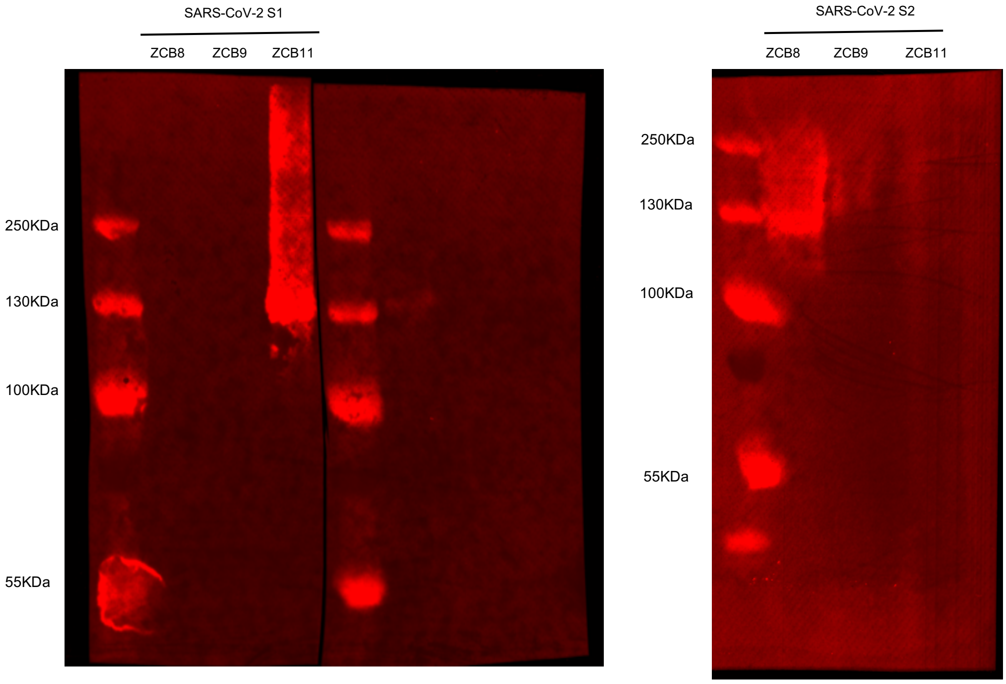

Supplement: Supplementary file 1 — Supplementary information [file 41467_2022_31259_MOESM1_ESM.pdf]
