# Supplementary material for: A broadly neutralizing antibody protects Syrian hamsters against SARS-CoV-2 Omicron challenge
Source: Nat Commun. 2022 Jun 23;13:3589. doi: 10.1038/s41467-022-31259-7 (PMC9223271; doi:10.1038/s41467-022-31259-7)
Supplement: Supplementary file 3 — Reporting Summary [file 41467_2022_31259_MOESM3_ESM.pdf]

nature portfolio

Corresponding author(s): Zhiwei Chen
Last updated by author(s): May 19, 2022

Reporting Summary

Nature Portfolio wishes to improve the reproducibility of the work that we publish. This form provides structure for consistency and transparency in reporting. For further information on Nature Portfolio policies, see our Editorial Policies and the Editorial Policy Checklist.

Statistics

For all statistical analyses, confirm that the following items are present in the figure legend, table legend, main text, or Methods section.

n/a	Confirmed	
☐	☒	The exact sample size (*n*) for each experimental group/condition, given as a discrete number and unit of measurement
☐	☒	A statement on whether measurements were taken from distinct samples or whether the same sample was measured repeatedly
☐	☒	The statistical test(s) used AND whether they are one- or two-sided<br>*Only common tests should be described solely by name; describe more complex techniques in the Methods section.*
☒	☐	A description of all covariates tested
☒	☐	A description of any assumptions or corrections, such as tests of normality and adjustment for multiple comparisons
☐	☒	A full description of the statistical parameters including central tendency (e.g. means) or other basic estimates (e.g. regression coefficient) AND variation (e.g. standard deviation) or associated estimates of uncertainty (e.g. confidence intervals)
☐	☒	For null hypothesis testing, the test statistic (e.g. *F*, *t*, *r*) with confidence intervals, effect sizes, degrees of freedom and *P* value noted<br>*Give P values as exact values whenever suitable.*
☒	☐	For Bayesian analysis, information on the choice of priors and Markov chain Monte Carlo settings
☒	☐	For hierarchical and complex designs, identification of the appropriate level for tests and full reporting of outcomes
☒	☐	Estimates of effect sizes (e.g. Cohen's *d*, Pearson's *r*), indicating how they were calculated

Our web collection on statistics for biologists contains articles on many of the points above.

Software and code

Policy information about availability of computer code

Data collection	Absorbance and luminescence were measured by SkanIt RE6.1 (ThermoScientific VARIOSKAN Lux).<br>Fluorescence images were scanned by Sapphire Capture Software V1.7.0319.0 (ThermoScientific azure biosystems)<br>qPCR data was collected by QuanStudio Real-Time PCR Software V1.3 (life technologies ViiA 7).<br>Flow cytometry was done by BD FACSAria III cell sorter (BD).<br>Antibody repertoire data was collected by NCBI/igblast tool suite (https://www.ncbi.nlm.nih.gov/igblast/).<br>SPR was performed by Biacore Control Software V3.2.1 (Cytiva, Biacore T200).<br>Cryo-EM data was collected by EPU software V2.10.0 with Titan Krios G3i (Thermo Fisher).<br>The lung sections with H&E staining were scanned by Akoya Vectra Polaris™ Automated Quantitative Pathology Imaging System.<br>Confocal images were captured by Carl Zeiss LSM 900.
Data analysis	Statistical analysis was done by Graphpad prism Version 8.0 or later.<br>Fluorescence intensity was analyzed using ImageJ or Fiji v1.53c (NIH).<br>Flow Cytometry data were analysed by FlowJo V10.<br>Antibody sequence alignment was performed by BioEdit V7.2.<br>Structure reconstruction was performed by cryoSPARC v 2.15.0 and RELION 3.1.<br>Atomic modeling was performed by UCSF Chimera v1.15, ChimeraX v1.3, Coot v0.9.5 and Phenix 1.20.<br>Antibody binding kinetics and competition analysis between antibodies were analyzed by Biacore Insight Evaluation Software v3.0 (Cytiva)<br>H&E images were processed by Phenochart 1.1.0<br>Confocal images were processed by ZEN 3.3 (blue edition)

For manuscripts utilizing custom algorithms or software that are central to the research but not yet described in published literature, software must be made available to editors and reviewers. We strongly encourage code deposition in a community repository (e.g. GitHub). See the Nature Portfolio guidelines for submitting code & software for further information.

Data

Policy information about availability of data

All manuscripts must include a data availability statement. This statement should provide the following information, where applicable:

- Accession codes, unique identifiers, or web links for publicly available datasets
- A description of any restrictions on data availability
- For clinical datasets or third party data, please ensure that the statement adheres to our policy

Data generated or analyzed during this study are included in this published article including supplementary information files. Source data are provided with this paper. We are applying for patent protection for some of the antibodies. All other information are also available from the corresponding author upon reasonable requests. Cryo-EM density maps of the spike-Fab complex have been deposited in the Electron Microscopy Data Bank (EMD-33195 for 3u, EMD-33194 for 2u1d, respectively). Atomic coordinates have been deposited in the Protein Data Bank with the accession code 7XH8 [https://www.rcsb.org/structure/unr-eleased/7XH8].

Field-specific reporting

Please select the one below that is the best fit for your research. If you are not sure, read the appropriate sections before making your selection.

- [x] Life sciences
- [ ] Behavioural & social sciences
- [ ] Ecological, evolutionary & environmental sciences

For a reference copy of the document with all sections, see nature.com/documents/nr-reporting-summary-flat.pdf

Life sciences study design

All studies must disclose on these points even when the disclosure is negative.

Sample size	The animal experiments was performed to give a n number of 4 or above. A n number equals to 4 is the the standard of biological experiments. No sample size calculation was performed. Sample size is chosen based on the standard of the corresponding field (PMID:32215622).
Data exclusions	No data was excluded.
Replication	All experiments were repeated in at least duplicates except indicated. Similar findings were obtained from all repeats.
Randomization	There was no allocation except for the grouping of the animals. Animals were randomly allocated to the groups.
Blinding	No blinding was done. Blinding was not relevant to the study because the results are quantitative and objective, and does not require a subjective judgment.

Reporting for specific materials, systems and methods

We require information from authors about some types of materials, experimental systems and methods used in many studies. Here, indicate whether each material, system or method listed is relevant to your study. If you are not sure if a list item applies to your research, read the appropriate section before selecting a response.

Materials & experimental systems

n/a	Involved in the study	
	x	Antibodies
	x	Eukaryotic cell lines
x		Palaeontology and archaeology
	x	Animals and other organisms
	x	Human research participants
x		Clinical data
x		Dual use research of concern

Methods

n/a	Involved in the study	
x		ChIP-seq
	x	Flow cytometry
x		MRI-based neuroimaging

Antibodies

Antibodies used	Goat anti-human IgG HRP (invitrogen #62-8420), 1:5000 for ELISA.<br>PE mouse anti-human IgG (BD Pharmingen #555787), 1ul/test for flow cytometry staining.<br>PB mouse anti-human CD3 (BD Pharmingen #558117), 1ul/test for flow cytometry staining.<br>PB mouse anti-human CD14 (Biolegend #325616), 1ul/test for flow cytometry staining.<br>PB mouse anti-human CD56 (Biolegend #318326), 1ul/test for flow cytometry staining.<br>PB mouse anti-human IgM (Biolegend #314514), 1ul/test for flow cytometry staining.

	PB mouse anti-human IgD (Biolegend #348224), 1ul/test for flow cytometry staining.<br>PerCP-Cy5.5 mouse anti-human CD19 (BD Pharmingen #561295), 1ul/test for flow cytometry staining.<br>PE/Cy7 mouse anti-human CD27 (Biolegend #356412), 1ul/test for flow cytometry staining.<br>DyLight 488 mouse anti-6X His Tag (Abcam #ab117512), 1ul/test for flow cytometry staining.<br>SureLight APC mouse anti-6X His Tag (Abcam #ab133714), 5ul/test for cytometry staining.<br>DyLight 650 mouse anti-6X His Tag (Abcam #ab117504), 1:1000 for western blot.<br>Rabbit anti-SARS-CoV-2 Nucleocapsid serum (In house production), 1:5000 for immunofluroscence.<br>AF488 goat anti-rabbit IgG (H+L) cross-absorbed secondary antibody (Life Technologies, #2110499), 1:1000 for immunofluorescence.
Validation	The in-house anti-SARS-CoV-2-NP immune serum were validated with ELISA, Western blots, and immunofluorescence staining in the previous publication (PMID: 32835326).<br>The commercial antibodies were only used for applications,with validation procedures described on the following sites of the manufacturers:<br>Goat anti-human IgG HRP:<br>https://www.thermofisher.com/antibody/product/Goat-anti-Human-IgG-Gamma-chain-Cross-Adsorbed-Secondary-Antibody-Polyclonal/62-8420<br>PE mouse anti-human IgG:<br>https://www.bdbiosciences.com/en-us/products/reagents/flow-cytometry-reagents/research-reagents/single-color-antibodies-ruo/<br>PB mouse anti-human CD3:<br>https://www.bdbiosciences.com/en-eu/products/reagents/flow-cytometry-reagents/research-reagents/single-color-antibodies-ruo/purified-mouse-anti-human-cd3.551916<br>PB mouse anti-human CD14:<br>https://www.biolegend.com/en-us/products/pacific-blue-anti-human-cd14-antibody-3957<br>PB mouse anti-human CD56:<br>https://www.biolegend.com/en-us/products/pacific-blue-anti-human-cd56-ncam-antibody-6679<br>PB mouse anti-human IgM:<br>https://www.biolegend.com/en-us/products/pacific-blue-anti-human-igm-antibody-6637<br>PB mouse anti-human IgD:<br>https://www.biolegend.com/en-us/products/pacific-blue-anti-human-igd-antibody-8205<br>PerCP-Cy5.5 mouse anti-human CD19:<br>https://www.bdbiosciences.com/en-nz/products/reagents/flow-cytometry-reagents/research-reagents/single-color-antibodies-ruo/percp-cy-5-5-mouse-anti-human-cd19.561295<br>PE/Cy7 mouse anti-human CD27:<br>https://www.biolegend.com/en-us/products/pe-cyanine7-anti-human-cd27-antibody-8640<br>DyLight 488 mouse anti-6X His Tag:<br>https://www.abcam.com/dylight-488-6x-his-tag-antibody-ad1110-ab117512.html<br>SureLight APC mouse anti-6X His Tag:<br>https://www.abcam.com/surelight-apc-6x-his-tag-antibody-ad1110-ab133714.html<br>DyLight 650 mouse anti-6X His Tag:<br>https://www.abcam.com/dylight-650-6x-his-tag-antibody-ad1110-ab117504.html<br>AF488 goat anti-rabbit IgG (H+L) cross-absorbed secondary antibody:<br>https://www.thermofisher.com/antibody/product/Goat-anti-Rabbit-IgG-H-L-Highly-Cross-Adsorbed-Secondary-Antibody-Polyclonal/A-11034

Eukaryotic cell lines

Policy information about cell lines

Cell line source(s)	HEK293T are from ATCC, Vero-E6-TMPRSS2, HEK293T-hACE2 are in house, and Expi293F cells are from Thermo Fisher Scientific.
Authentication	All cell lines were frequently checked for cellular morphologies, growth rates and functions. All purchased cell lines were available in commercial company.
Mycoplasma contamination	All cell lines were tested negative for mycoplasma contamination.
Commonly misidentified lines (See ICLAC register)	No commonly misidentified cell lines were used.

Animals and other organisms

Policy information about studies involving animals; ARRIVE guidelines recommended for reporting animal research

Laboratory animals	6-10 week old male and female golden Syrian hamsters were used in this study, which has been specified in the figure legends.
Wild animals	No wild animals were used in the study.
Field-collected samples	No field collected samples were used in the study.

Ethics oversight	All animal studies were approved by the Committee on the Use of Live Animals in Teaching and Research (CULATR 5359-20 and 5518-20), the University of Hong Kong.

Note that full information on the approval of the study protocol must also be provided in the manuscript.

Human research participants

Policy information about studies involving human research participants

Population characteristics	The study enrolled a total of 34 donors aged 20 to 66 years old who received two doses of BNT162b2 mRNA vaccines before June 2021.
Recruitment	Study participants were recruited on the voluntary basis from vaccinated donors. The exclusion criteria include individuals with (1) documented SARS-CoV-2 infection, (2) high-risk infection history within 14 days before vaccination, (3) COVID-19 symptoms such as sore throat, fever, cough and shortness of breath. Total 34 of vaccinated donors were finally selected.
Ethics oversight	This acquisition of blood samples from vaccinated donors for identification of broad neutralizing activities and isolation of potent monoclonal antibodies against COVID-19 received approval from the Institutional Review Board of The University of Hong Kong/Hospital Authority Hong Kong West Cluster (Ref No. UW 21-120 452). The research was conducted in strict accordance with the rules and regulations of the Hong Kong government for the protection of human subjects. The study subjects agreed and signed the written informed consents for research use of their blood samples. All data was analysed anonymously with indirect identifiers.

Note that full information on the approval of the study protocol must also be provided in the manuscript.

Flow Cytometry

Plots

Confirm that:

☒ The axis labels state the marker and fluorochrome used (e.g. CD4-FITC).

☒ The axis scales are clearly visible. Include numbers along axes only for bottom left plot of group (a 'group' is an analysis of identical markers).

☒ All plots are contour plots with outliers or pseudocolor plots.

☒ A numerical value for number of cells or percentage (with statistics) is provided.

Methodology

Sample preparation	PBMCs from the blood of vaccinated donors were isolated and then resuspended in FACS buffer (PBS containing 2% FBS). The resuspended PBMCs were stained with an antibody cocktail and a His-tagged SARS-CoV-2 spike protein for 30 min at 4 °C for identification of SARS-CoV-2 antigen-specific memory B cells. After washes with FACS buffer, cells went through cell strainer (BD) before cell sorting.
Instrument	BD FACSAria III cell sorter
Software	FlowJo V10
Cell population abundance	The number of SARS-CoV-2 spike specific IgG+ memory B cells accounted for 0.93% of the total IgG+ memory B cells. The sorted target population were collected and then re-run to determine the purity of the target population.
Gating strategy	The PBMCs were gated with FSC-A and SSC-A for lymphocytes, and then gated with FSC-A and FSC-H for singlets, and then gated with Zombie and CD3,CD14, CD56, IgM, IgD for IgM-IgD- B cells for live B cells, followed by CD19 and FSC-A for specific B cells, and then gated with CD27 and IgG for IgG+ memory B cells, and then gated with APC and FITC-conjugated anti-his-spike probes for SARS-CoV-2 spike specific IgG+ memory B cells.

☒ Tick this box to confirm that a figure exemplifying the gating strategy is provided in the Supplementary Information.
